# User Retention and Engagement in the Digital-Based Diabetes Education and Self-Management for Ongoing and Newly Diagnosed (myDESMOND) Program: Descriptive Longitudinal Study

**DOI:** 10.2196/44943

**Published:** 2023-07-21

**Authors:** Mary M Barker, Radhika Chauhan, Melanie J Davies, Christopher Brough, Alison Northern, Bernie Stribling, Sally Schreder, Kamlesh Khunti, Michelle Hadjiconstantinou

**Affiliations:** 1 Diabetes Research Centre University of Leicester NIHR Leicester Biomedical Research Centre Leicester United Kingdom; 2 Institute of Environmental Medicine karolinska institutet Stockholm Sweden; 3 Leicester Diabetes Centre University Leicester Hospitals Trust Leicester United Kingdom

**Keywords:** retention, engagement, digital self-management, type 2 diabetes, mobile phone

## Abstract

**Background:**

Digital health interventions have the potential to improve the physical and psychosocial health of people living with type 2 diabetes. However, research investigating the long-term (≥1 year) retention and engagement of users within these programs is limited.

**Objective:**

The aim of this study was to evaluate long-term user retention and engagement in the digital-based Diabetes Education and Self-Management for Ongoing and Newly Diagnosed (myDESMOND) program, using real-world data.

**Methods:**

Anonymized data from all myDESMOND users who registered with the program on or before November 16, 2020, were included in the analyses. User retention was defined as the period between the day a user registered with the myDESMOND program and their last day of access. The primary engagement outcome was defined as the total number of log-ins to the program per user. The associations between retention, engagement, and sociodemographic factors (age, sex, and ethnicity) were tested using Cox regression models and Wilcoxon rank sum tests.

**Results:**

A total of 9522 myDESMOND users were included in this analysis. Of the 9522 users, 5360 (56.29%) remained on the program for at least a month, whereas 1676 (17.6%) remained on the program for at least 1 year. Retention was significantly higher among older users; the adjusted hazard ratio (representing the risk of users leaving the program within the first year) among users aged ≥50 years, compared with those aged <50 years, was 0.79 (95% CI 0.75-0.84; *P*<.001). The median number of myDESMOND log-ins per user was 8 (IQR 4-8); however, this was significantly lower among users aged <50 years (*P<*.001). Engagement metrics also differed according to sociodemographic characteristics; the estimated time spent per log-in was 5.35 (IQR 2.22-11.80) minutes among all users; however, this was significantly higher among female users (*P*<.001), users aged ≥50 years (*P*<.001), and users of White ethnicity (*P*=.02).

**Conclusions:**

Although retention and engagement of users within myDESMOND were found to be high, these findings highlight the need for age- and culture-specific implementation strategies and content adaptations to improve retention and engagement among all users of self-management programs.

## Introduction

### Background

Recent figures published by the International Diabetes Federation reported that an estimated 463 million individuals were affected by diabetes in 2019, with 90% of them constituting people with type 2 diabetes (T2D) [1]. It is anticipated that this global prevalence will increase to 578 million by 2030 and 700 million by 2045 [1]. T2D often leads to serious microvascular (neuropathy, nephropathy, and retinopathy) and cardiovascular complications, with the latter representing a major cause of comorbidity and mortality among this population [2-4]. Globally recognized as an essential component of T2D care [5], diabetes self-management education and support (DSMES) has been found to be highly cost-effective, reduce the developmental risk of health complications, and increase the well-being of individuals with T2D [6-8].

Despite significant clinical, psychological, and behavioral benefits, DSMES programs remain largely underused, with a significant proportion of the population with diabetes opting not to attend [9]. In 2020, data published by the National Diabetes Audit revealed that only 5.6% of the adults living with T2D in the United Kingdom attended a structured DSMES program within 12 months of their diagnosis [10]. Qualitative studies have identified several barriers contributing to the low uptake of traditional face-to-face structured DSMES programs, including physical and psychosocial comorbidities, a lack of accessibility, competing priorities (family and work), and diabetes-related shame and stigma [11-13]. Digital DSMES programs have the potential to overcome many of these barriers [14] and, in recent years, have become increasingly integrated into T2D care [6].

Digital-based Diabetes Education and Self-Management for Ongoing and Newly Diagnosed (myDESMOND) [6], HeLP-Diabetes [15], the Low Carb Program [16], Patient-Centered Smartphone-Based Diabetes Care System [17], GlycoLeap [18], and GlucoNote [19] are some of the many digital or smartphone-based programs that have been tested for people living with T2D. Many of these programs, including myDESMOND [6], Healthy Living [20], the Low Carb Program [16], and GlycoLeap [18], have now become available to the wider public. Such DSMES programs have shown favorable results [5], with a meta-analysis of 14 randomized controlled trials evaluating digital self-management apps reporting a pooled mean reduction of −0.49% in glycated hemoglobin levels among T2D participants [21].

Despite promising outcomes, digital DSMES programs can suffer from low user retention and engagement [22-24], meaning that users are not able to fully experience the clinical and psychosocial benefits [25]. The evaluation of user retention and engagement as well as factors affecting retention (eg, participant demographics) [26] has the potential to highlight important indicators of real-world implementation barriers with regard to digital health programs [27], thus facilitating the development of informed and targeted retention strategies [26]. Nonetheless, few digital DSMES programs have evaluated such data, with the findings focusing predominantly on clinical and cognitive impact as well as usability [28].

### Objectives

Current evidence surrounding user retention and engagement for people with T2D is limited to the following digital self-management programs: HeLP-Diabetes [15], My Care Hub [29], and GlucoNote [19]. These studies have reported conflicting findings, with 1-month retention rates varying from 9% (HeLP-Diabetes) [15] to 35.3% (GlucoNote) [19]. Furthermore, with both HeLP-Diabetes [15] and My Care Hub [29] evaluating data after short-term intervention periods of 4 weeks and 3 weeks, respectively, there is scarce information available regarding long-term retention and engagement in the existing digital literature. Long-term retention data are limited to a study in Japan by Yamaguchi et al [19], who analyzed retention rates across a 1-year period for 357 participants with access to GlucoNote. The findings from this study revealed an overall decrease in long-term retention, with rates reducing from 35.3% (at 1 month) to 22% (at 3 months) [19]. With Yamaguchi et al [19] focusing on user retention in a predominantly male (79.9%) participant group, it is clear that there is need for a better understanding of both long-term (≥1 year) user retention and engagement across a larger population-based sample of people living with T2D. Thus, this paper aimed to investigate long-term retention and engagement, in addition to associated factors, among >9000 users of myDESMOND.

## Methods

### The myDESMOND Program

The myDESMOND program, developed by a multidisciplinary team at the Leicester Diabetes Centre in Leicester, United Kingdom, and launched in 2018, is a digital self-management education program based on Diabetes Education and Self-Management for Ongoing and Newly Diagnosed (DESMOND), an evidence- and theory-based group education program for people living with T2D [30-32]. myDESMOND can be freely accessed via smartphones, tablet devices, laptops, and desktop computers and was developed using an iterative approach based on optimizing the learning and engagement of users [6]. Multiple core functions are available in the myDESMOND program, including interactive learning sessions; weekly booster sessions building on the topics covered in the learning sessions; health and activity trackers; and the *Decision Maker* tool, which allows users to set goals to improve their health. myDESMOND also offers other social features, such as the *Ask the Expert* function that allows users to seek advice and guidance from Leicester Diabetes Centre’s multidisciplinary team, a chat feature whereby users can interact with other users in the myDESMOND community, and an innovative *Buddies* function that allows users to invite up to 5 family members or friends to join them in their myDESMOND journey and compete with them in weekly or daily activity challenges. myDESMOND is part of routine care at 90 health care organizations across the United Kingdom and Ireland, and individuals participating in DESMOND are usually signposted to the program as an ad hoc resource. myDESMOND users have access to 10 weeks of booster sessions, but they can also have access for life if they want.

### Data

With users’ consent, demographic and use data are collected for all users and stored on an encrypted server. Anonymized data can subsequently be downloaded for analysis.

### Ethical Considerations

As this study presents a service evaluation, no specific ethics approval was needed; however, all users of myDESMOND have agreed to the terms and conditions of the privacy policy before they use the program. This policy includes a statement regarding use of their anonymized data for service evaluations.

### Study Population

Since myDESMOND was launched in 2018, a total of 21,285 users have registered with the program. Data were extracted on November 16, 2021. For user retention to be analyzed over a full year, only users registered with the program on or before November 16, 2020, were included in the analysis, meaning that all included users had at least 1 full year of data.

### Variables

User retention was defined as the duration of time between the day a user registered with the myDESMOND program and the last day that they accessed the program. The primary user engagement outcome was the total number of log-ins per user. The following secondary user engagement outcomes were also analyzed:

- Total time spent using the program per user
- Estimated time spent per log-in (calculated as the total time spent in the program divided by the total number of log-ins, per user)
- Log-ins per week (calculated as the total number of log-ins divided by the number of weeks spent using the program, per user)

Data were also available for users’ sex, age, and ethnicity.

### Statistical Analysis

Sociodemographic variables (sex, age, and ethnicity) were summarized using median (IQR) or frequency (percentage), as appropriate. Age was used as a categorical variable (<50 years or ≥50 years). The effect of an alternative categorization of age (<40 years or ≥40 years) on the findings was explored in a supplementary analysis. Ethnicity was categorized as White, Black, Asian, other, or mixed. Because of the small number of users categorized as other or mixed ethnicity, only the White and Black or Asian ethnic groups were included in the analysis. Survival analysis was conducted to investigate the retention of users in the myDESMOND program during their first year of registration. Kaplan-Meier curves were generated for all users and stratified by age, sex, and ethnicity. As the assumption of proportional hazards was not violated, Cox regression models were subsequently run to estimate the hazard ratios of users leaving the myDESMOND program by sex, age group, and ethnicity. Both univariate and multivariable models were run, adjusted for sex, age group, and ethnicity, as appropriate. Complete case analysis was used throughout. Previous research has shown a substantial difference in overall program retention when users who left the program after <1 day were excluded from the analysis compared with analysis undertaken using data from all users [26]. Therefore, a further supplementary analysis was conducted that excluded users who spent <1 day on the program.

The primary and secondary user engagement variables were evaluated over the total duration of program use, which could range from <1 day to >1 year. The total number of log-ins and total time spent in the program were first summarized by calculating the median (IQR) of these metrics, stratified by duration in the program. All engagement metrics were then summarized, stratified by sex, age group, and ethnicity. As the data did not follow a normal distribution, Wilcoxon rank sum tests were conducted to investigate any differences in the engagement metrics by sex, age group, or ethnicity. The analysis was conducted in Stata (version 17.0; StataCorp LLC). Statistical significance was set at *P*<.05 throughout.

## Results

### Sociodemographic Characteristics of Users

This analysis included 9522 users of the myDESMOND program, of whom 3974 (41.73%) were male and 3843 (40.36%) were female. The median age of these users was 59 (IQR 51-68) years. Of the 9522 users, 532 (5.59%) were aged <40 years, whereas 1697 (17.82%) were aged <50 years, and 6135 (64.43%) were aged ≥50 years. The majority of the users (6478/9522, 68.03%) were White, whereas 11.96% (1139/9522) were Black or Asian, 1.79% (171/9522) reported an ethnicity classified as other or mixed, and 18.21% (1734/9522) had missing ethnicity data (Table 1). These sociodemographic characteristics were similar when users who spent <1 day using the myDESMOND program were excluded (Table S1 in Multimedia Appendix 1). Stratification of the age and sex variables by ethnicity showed that the median age in the Black or Asian ethnicity group (51, IQR 43-59, years) was far lower than that observed in the White ethnicity group (61, IQR 53-69, years; Table S2 in Multimedia Appendix 1).

**Table 1:** Sociodemographic characteristics (N=9522).

			Values
**Sex, n (%)**			
	Male	3974 (41.73)	
	Female	3843 (40.36)	
	Missing	1705 (17.91)	
Age (years; n=7832), median (IQR)			59 (51-68)
**Age (categorization: <40 years or ≥40 years), n (%)**			
	<40	532 (5.59)	
	≥40	7300 (76.66)	
	Missing	1690 (17.75)	
**Age (categorization: <50 years or ≥50 years), n (%)**			
	<50	1697 (17.82)	
	≥50	6135 (64.43)	
	Missing	1690 (17.75)	
**Ethnicity, n (%)**			
	White	6478 (68.03)	
	Black or Asian	1139 (11.96)	
	Other or mixed	171 (1.79)	
	Missing	1734 (18.21)	

### User Retention

**Figure 1:**
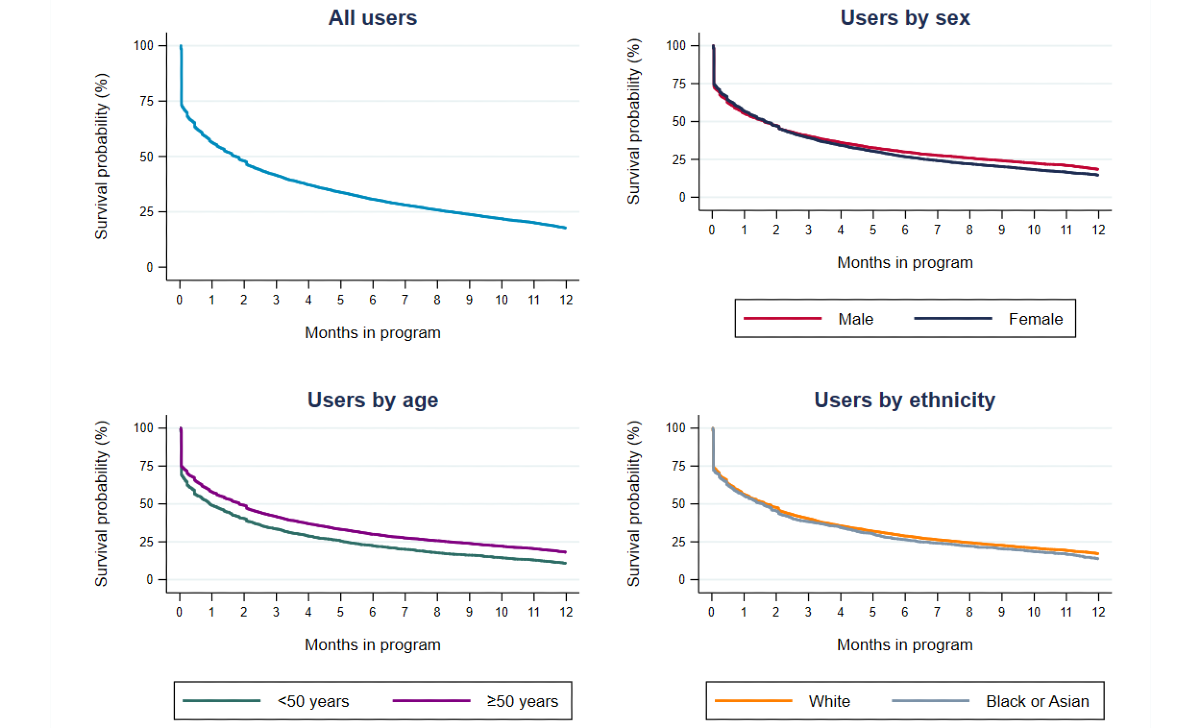
Kaplan-Meier curves showing the time to users stopping use of the digital-based Diabetes Education and Self-Management for Ongoing and Newly Diagnosed app after the course of a year for all users, stratified by sex, age, and ethnicity.

The duration that users remained on the myDESMOND program ranged from <1 day to 40.4 months (3.4 years), with a median of 7.57 (IQR 0.00-36.43) weeks. Of the 9522 users, 5360 (56.29%) used the myDESMOND program for at least 1 month, 2914 (30.6%) used the program for at least 6 months, and 1676 (17.6%) remained on the program for at least 1 year (Figure 1).

When users who spent <1 day using myDESMOND were excluded from the analysis, 75.5% (5360/7099) of the users spent at least 1 month using the program (Figure S1 in Multimedia Appendix 1). Figure 1 displays survival curves stratified by sex, age group, and ethnicity. In both the univariate and multivariable analyses, older age was significantly associated with a lower likelihood of leaving the program during the analysis period. Corresponding adjusted hazard ratios were 0.79 (95% CI 0.75-0.84; *P*<.001) for users aged ≥50 years compared with those aged <50 years, and 0.77 (95% CI 0.70-0.85; *P*<.001) for users aged ≥40 years compared with those aged <40 years (Table 2; Table S3 and Figure S2 in Multimedia Appendix 1). Although the median duration of time spent in the program was slightly longer for female users compared with male users, female users had a significantly higher likelihood of leaving the program within the year in both the univariate (*P*=.003) and multivariable analyses (*P*=.03). No significant associations were observed between ethnicity and the likelihood of users leaving the program (Table 2). Similar results were observed when users who spent <1 day using the program were excluded from the analysis (Table S4 in Multimedia Appendix 1).

**Table 2:** Results from Cox proportional hazard models reporting associations between sex, age, ethnicity, and survival time in the program.

		Unadjusted model			Adjusted model	
		Hazard ratio (95% CI)	*P* value	Hazard ratio (95% CI)		*P* value
**Sex**						
	Male	1.00 (reference)	N/A^a^	1.00 (reference)		N/A
	Female	1.08 (1.03-1.13)	.003	1.06 (1.01-1.11)		.03
**Age**						
	<50 years	1.00 (reference)	N/A	1.00 (reference)		N/A
	≥50 years	0.79 (0.75-0.84)^b^	<.001	0.80 (0.75-0.85)		<.001
**Ethnicity**						
	White	1.00 (reference)	N/A	1.00 (reference)		N/A
	Black or Asian	1.08 (1.01-1.15)^c^	.03	1.01 (0.94-1.09)		.72

^a^N/A: not applicable.

^b^Sex, age, and ethnicity (as appropriate) included as confounders to generate adjusted hazard ratios.

^c^Includes users with nonmissing age and sex data, as well as users classified as White, Black, or Asian.

### User Engagement: Primary Outcome

On average, users logged into the myDESMOND program 8 (IQR 4-18) times during their duration of myDESMOND use, which ranged from <1 day to 40.4 months. However, the total number of log-ins per user was significantly lower among younger users (*P*<.001), as well as among those from a Black or Asian ethnic background (*P*=.01; Table 3; Table S5 in Multimedia Appendix 1).

**Table 3:** Retention and engagement metrics by sex, age, and ethnicity^a^.

		Duration in the program (weeks)	Total number of log-ins	Total time spent using program (minutes)	Estimated time spent per log-in (minutes)	Log-ins per week
Total, median (IQR)		7.57 (0.00-36.43)	8 (4-18)	63.74 (20.87-191.80)	5.35 (2.22-11.80)	0.77 (0.32-1.84)
**Sex**						
	Male, median (IQR)	7.00 (0.00-36.86)	8 (4-20)	75.74 (24.45-221.17)	5.82 (2.60-12.57)	0.80 (0.35-1.89)
	Female, median (IQR)	7.14 (0.14-28.57)	8 (5-18)	82.55 (28.80-232.43)	6.82 (3.07-14.00)	0.89 (0.41-1.93)
	*P* value^b^	.37	.76	.02	<.001	.06
**Age**						
	<50 years, median (IQR)	4.00 (0.00-22.14)	7 (4-15)	62.33 (20.33-161.58)	5.53 (2.35-12.38)	0.88 (0.36-2.17)
	≥50 years, median (IQR)	8.00 (0.29-36.29)	9 (5-20)	86.10 (28.53-245.23)	6.53 (2.98-13.64)	0.84 (0.38-1.87)
	*P* value	<.001	<.001	<.001	<.001	.13
**Ethnicity**						
	White, median (IQR)	7.14 (0.14-33.43)	9 (5-19)	81.75 (28.0-227.85)	6.40 (2.88-13.53)	0.86 (0.38-1.97)
	Black or Asian, median (IQR)	6.86 (0.00-28.14)	7 (4-17)	70.40 (20.85-223.33)	5.80 (2.50-13.28)	0.80 (0.35-1.75)
	*P* value	.07	.005	.01	.02	.05

^a^Excludes users who spent <1 week using the web-based Diabetes Education and Self-Management for Ongoing and Newly Diagnosed program.

^b^*P* values were calculated using Wilcoxon rank sum tests.

### User Engagement: Secondary Outcomes

Users spent a median total of 63.74 (IQR 20.87-191.80) minutes using the program. Younger users (*P*<.001), male users (*P*=.02), and Black or Asian users (*P*=.01) spent significantly less time, in total, using the program. On average, users spent 5.35 (IQR 2.22-11.80) minutes in the program per log-in. However, this metric was significantly lower for male users (*P*<.001), younger users (*P*<.001), and Black or Asian users (*P*=.02). The median number of log-ins per week was 0.77 (IQR 0.32-1.84) for all users included in the analysis (Table 3; Table S5 in Multimedia Appendix 1).

Users who spent ≤3 months using the myDESMOND program had an average of 5 (IQR 4-8) log-ins and spent a total of 39.40 (IQR 14.68-97.57) minutes using the program. These engagement metrics increased to 15 (IQR 8-30) log-ins and 152.17 (95% CI 64.79-355.93) minutes using the program among users who spent >9 months using myDESMOND (Table 4).

**Table 4:** Total number of log-ins and total time spent in the program, stratified by duration in the program.

Duration in the program (months)	Total number of log-ins, median (IQR)	Total time spent in the program (minutes), median (IQR)
≤3	5 (4-8)	39.40 (14.68-97.57)
>3 to 6	11 (6-19)	105.67 (36.05-252.27)
>6 to 9	14 (7-26)	132.68 (48.80-307.66)
>9 to 12	15 (8-30)	152.17 (64.79-355.93)

## Discussion

### Overview

To date, research evaluating long-term user retention and engagement with digital DSMES programs among adults with T2D has been limited, with long-term data focusing exclusively on retention in a small non–population-based sample of people with both T2D and prediabetes [19]. This is the first study to investigate both long-term retention and engagement (and associated factors) for a digital diabetes self-management program across a large and ethnically diverse sample of adults living with T2D. Our findings demonstrated high levels of user retention and engagement, which differed significantly according to sociodemographic characteristics.

### User Retention

In comparison with previously reported 1-month retention rates of 9% (HeLP-Diabetes) [15] and 35.3% (GlucoNote) [19], our findings showed a favorable retention rate of 56% at 1 month among myDESMOND users. Furthermore, in contrast to the 3-month retention rate of 22% after 1 year’s access to GlucoNote in the study by Yamaguchi et al [19], our retention rate of 31% over 6 months compares much more favorably, thus highlighting the potential of the myDESMOND program at sustaining long-term retention among users. myDESMOND offers quality educational content that is accompanied by a wide breadth of functionalities (booster sessions, health and activity trackers, *Ask the Expert*, chat feature, etc), meaning that the program has potential to accommodate a variety of user-specific needs. Further research looking at ways to investigate program-specific functionalities associated with higher retention rates would be useful to facilitate the design of future digital self-management programs. Furthermore, it is important to note that some of the myDESMOND users would have attended the DESMOND group self-management program before registering, meaning that they already had an insight into the quality of the educational content and functionalities available. This suggests that information provision and supplementary group self-management programs may be a suitable way to encourage user retention across digital self-management programs.

Despite promising user retention rates, discrepancies in the existing definitions of retention make direct comparisons of our findings challenging. As our study was conducted using real-world data, we defined retention as the duration of time between the day a user registered with the myDESMOND program and their last day accessing the program; however, other studies have largely defined retention as the completion of a postintervention assessment [15,29]. It is clear that there is need for a standardized definition of retention that also takes into account the real-world application of digital self-management programs.

Our analysis also showed a median retention duration of 7.57 weeks, which is substantially greater than the 8-day median retention reported by Yamaguchi et al [19]. Furthermore, our findings also revealed both categorizations of age (40 years and 50 years) to be significantly associated with program retention in both the univariate and multivariable analyses (adjusting for sex and ethnicity), with younger users showing a significantly higher likelihood of leaving myDESMOND and significantly lower duration of use of the program than older users. Similar findings have also been reported by other digital health studies exploring retention indicators in long-term health conditions in addition to diabetes [26]. The 2 categorizations of age were used to observe whether retention differed in users with early-onset T2D versus those with usual-onset T2D. However, as the proportion of users aged <40 years was very low, the categorization of <50 years was used in the main analysis to maintain power, with the alternative categorization of <40 years being explored in the supplementary analysis.

Our study is not the first to report low retention among young users of a digital diabetes self-management program. A randomized controlled trial evaluating use of the Young with Diabetes app among young people with type 1 diabetes in Denmark also reported poor retention, with app use decreasing rapidly to a retention rate of 5% at 12 months [33]. According to Klasnja et al [34], after the initial diagnosis period, people with diabetes develop flexible self-management routines, with their focus shifting to quality of life; hence, their use of diabetes health technology may fluctuate accordingly with periods of infrequent use. On the basis of our findings and the existing literature [33,34], it is feasible to suggest that, for young people with diabetes, maintaining a high quality of life may involve focusing on other aspects of their lives, such as education, employment, independent living, and families, thus reducing their regular use of a digital self-management program that may not adequately address their presenting need or concern. Further research exploring how to adapt digital self-management programs to address these periods of infrequent or intermittent use by young people with diabetes may be a crucial step toward the development of age-specific retention strategies for this cohort.

### User Engagement

Our findings revealed that myDESMOND users spent an average total of 63.74 minutes on the program, with an average of 5.35 minutes spent in the program per log-in. From a behavioral perspective, engagement with digital behavior change interventions has largely been defined as *use*, with a focus on rate, duration, and depth of use, in addition to associated factors [35,36]. Consistent with this definition, our study reported the number of user log-ins and time spent in the program per log-in (and associated factors) to evaluate user engagement. However, unlike our study, the studies conducted by Glasgow et al [37] and Adu et al [29] reported a wider range of use metrics to capture the multidimensional nature of user engagement [36,38]. Adu et al [29] explored user engagement using a modified version of the frequency, intensity, time, and type (FITT) principle, thus acknowledging the frequency (how often the user visits the app or intervention), intensity (depth of engagement; eg, number of app or intervention features used out of those available), time (length of use during a single visit to the app or intervention), and type (eg, reflective [self-reporting of behavior or health outcomes] or didactic [reading posts and completing quizzes or challenges] engagement) of engagement with the My Care Hub app [29,38,39]. The FITT principle has the potential to effectively capture all domains of use data relating to the behavioral conceptualization of engagement [38]. Therefore, further evaluation of engagement with the myDESMOND program and other digital programs using the FITT principle is recommended because this may allow investigation of the intensity and type of engagement across programs. Such evaluations may provide new insights into the appropriateness of this principle in the analysis of real-world use data, hence contributing to the development of an all-encompassing universal measure of engagement.

Our findings revealed age to be significantly associated with engagement with the myDESMOND program. In comparison with older users, younger users had a significantly lower median number of log-ins and average time spent in the program per log-in. The observed age-related differences in engagement may be attributable to the lack of age-appropriate content and functionality for younger users. Previous research has emphasized the need for the provision of tailored age-specific education, information, and peer support for young people with T2D to promote effective self-management of their condition [40-42]. Despite this, few age-appropriate digital self-management programs for T2D currently exist. Previous studies investigating the effectiveness of digital self-management programs tailored for young people with type 1 diabetes have shown potential for enhancing daily self-management through the emotional and social support benefits associated with the provision of an age-specific online peer support element [43,44]. More specifically, having a web-based platform to share and discuss personal experiences with other individuals of a similar age was found to reduce feelings of loneliness and isolation, thus motivating individuals to implement daily self-management behaviors [43,44]. Furthermore, in both studies, the peer support element remained the most consistently and frequently used functionality across both self-management programs [43,44]. Therefore, it is possible that adapting the existing myDESMOND chat function to allow age-specific interactions may facilitate increased engagement with the program among younger users, as observed in the previously mentioned studies [43,44].

In line with previous research [45], our analysis also showed reduced engagement with the myDESMOND program among ethnic minority groups with T2D, with users of Black or Asian ethnicity showing significantly lower average number of log-ins and time spent in the program per log-in. Previous studies investigating the effectiveness of culturally tailored diabetes self-management education programs for ethnic minority groups have reported multifaceted benefits, with significant improvements shown across a range of clinical, knowledge, and psychobehavioral outcomes [46-48]. Although predominantly exploring face-to-face and telecommunication interventions or programs, these studies emphasize the importance of considering cultural and linguistic differences to promote increased uptake and overall health benefits among ethnic minority groups. Furthermore, Yardley et al [35] highlighted that a user’s engagement with a digital behavior change intervention can be sustained, reduced, or molded by sociocontextual influences, including their wider cultural setting. Therefore, it is feasible to suggest that cultural adaptation of myDESMOND to meet the needs of ethnic minority groups with T2D may promote their increased engagement with the program. In addition, addressing existing ethnic disparities in digital health care [49,50], including accessibility to digital platforms such as myDESMOND, may also promote increased engagement among this cohort.

It is important to note, however, that the associations observed between ethnicity and user engagement may be confounded by age; users of Black or Asian ethnicity were substantially younger than users of White ethnicity.

Similar to age and ethnicity, sex was also revealed by our findings to be significantly associated with engagement with the myDESMOND program, with male users spending significantly less time in the program per log-in compared with female users. This is consistent with previous literature reporting significantly greater engagement in digital health care among female users [51]. Although limited literature exists regarding sex differences in engagement with digital diabetes self-management programs, it is well known that sex is a crucial characteristic affecting optimal diabetes self-management [52], with male individuals and female individuals living with T2D experiencing differing biological, psychological, and physical needs and challenges [53]. Female individuals with T2D have been found to experience less favorable long-term physical and mental health outcomes; yet, they have been known to exhibit better self-management behaviors than their male counterparts [54]. Consequently, it is unsurprising that research has frequently emphasized the need for sex-specific diabetes care and support to improve long-term health outcomes among both cohorts [54,55]. Although most research pertaining to sex-specific diabetes support has focused on female individuals and looked at routine self-management [52], our findings highlight the need for research exploring the unique sex-related barriers and challenges contributing to long-term engagement with digital diabetes self-management programs. Such research has the potential to highlight important aspects of sex-specific content and functionality that may enhance engagement with programs such as myDESMOND in both male and female users, thereby contributing to long-term improvements in health outcomes among both male and female individuals with T2D.

### Strengths and Limitations

This analysis has many strengths; namely, it used real-world data from >9000 users, thereby capturing a highly valid picture of the retention and engagement of myDESMOND users. The analysis was limited by the inability to differentiate between changes in use over the time spent in the program and different types of myDESMOND use (eg, learning sessions vs social features), which would have allowed for a better understanding of the differences in retention and engagement in specific program features across users. However, the analysis of retention, various engagement metrics, and multiple potentially associated sociodemographic factors provided a comprehensive understanding of the differing retention and engagement levels observed among myDESMOND users. Future research should investigate the impact of increased engagement and retention on clinical outcomes, such as glycated hemoglobin levels. Finally, although the multiethnic nature of the sample allowed for a thorough investigation of the association between ethnicity and retention or engagement, a relatively high proportion of users had missing data for ethnicity and other sociodemographic variables, meaning that not all participants could be included in this analysis, potentially resulting in a lack of power in some supplementary analyses.

### Conclusions

This study explored long-term retention and engagement among >9000 users of the myDESMOND program, finding a higher retention rate than has previously been reported from analyses of other digital T2D self-management programs. The levels of engagement with myDESMOND were also promising. Further analysis investigating engagement by type of use is required. In addition, the myDESMOND program would benefit from age- and culture-specific adaptations to improve the engagement of all users.

## Appendix

Multimedia Appendix 1 Sociodemographic characteristics, association between variables, and retention and engagement metrics by age.

## Abbreviations

DESMOND: Diabetes Education and Self-Management for Ongoing and Newly Diagnosed
DSMES: diabetes self-management education and support
FITT: frequency, intensity, time, and type
myDESMOND: digital-based Diabetes Education and Self-Management for Ongoing and Newly Diagnosed
T2D: type 2 diabetes

## Data Availability

The data that support the findings of this study are available from the Leicester Diabetes Centre, but restrictions apply to the availability of these data, which were used under license for this study; therefore, the data are not publicly available.
