# Supplementary material for: User Retention and Engagement in the Digital-Based Diabetes Education and Self-Management for Ongoing and Newly Diagnosed (myDESMOND) Program: Descriptive Longitudinal Study
Source: JMIR Diabetes. 2023 Jul 21;8:e44943. doi: 10.2196/44943 (PMC10403792; doi:10.2196/44943)
Supplement: Multimedia Appendix 1 [file diabetes_v8i1e44943_app1.docx]

**Supplementary Material**

**Supplementary Table 1:** Sociodemographic characteristics, excluding users who spent <1 day in the programme

|  | **Total (n=7,099)** |
| --- | --- |
| **Sex** | |
| Male | 2,971 (41.9) |
| Female | 2,921 (41.1) |
| Missing | 1,207 (17.0) |
| **Age** |  |
| Median (IQR)^a^ | 60 (51-68) |
| <40 years | 368 (5.1) |
| ≥40 years | 5,535 (78.0) |
| <50 years | 1,217 (17.1) |
| ≥50 years | 4,686 (66.0) |
| Missing | 1,196 (16.9) |
| **Ethnicity** | |
| White | 4,920 (69.3) |
| Black/Asian | 838 (11.8) |
| Other/Mixed | 116 (1.6) |
| Missing | 1,225 (17.3) |

^a^N=5,903

Data presented as number (%), unless otherwise indicated

**Supplementary Table 2:** Age and sex variables, stratified by ethnicity

|  | **Median (IQR)** | | |
| --- | --- | --- | --- |
|  | **White** (n=6,478) | **Black/Asian** (n=1,139) | **Other/Mixed** (n=171) |
| **Sex** | | | |
| Male | 3,294 (50.8) | 585 (51.3) | 74 (43.3) |
| Female | 3,173 (49.0) | 551 (48.4) | 97 (56.7) |
| Missing | 11 (0.2) | 3 (0.3) | 0 (0) |
| **Age** | | | |
| Median (IQR)^a^ | 61 (53-69) | 51 (43-59) | 53 (45-61) |
| <40 years | 335 (5.2) | 168 (14.7) | 26 (15.2) |
| ≥40 years | 6,143 (94.8) | 971 (85.3) | 145 (84.8) |
| Missing | 0 (0) | 0 (0) | 0 (0.0) |
| <50 years | 1,124 (17.4) | 499 (43.8) | 63 (36.8) |
| ≥50 years | 5,354 (82.6) | 640 (56.2) | 108 (63.2) |
| Missing | 0 (0) | 0 (0) | 0 (0.0) |

^a^N=5,903

**Supplementary Table 3:** Results from cox proportional hazard models reporting associations between age (categorised as <40 years or ≥ 40 years) and survival time in programme

|  | **Unadjusted Model** | | **Adjusted Model** | |
| --- | --- | --- | --- | --- |
|  | **Hazards Ratio (95% CI)** | ***P* value** | **Hazards Ratio (95% CI)** | ***P* value** |
| **Age** | |  |  |  |
| <40 years | 1.00 (ref) | <.001 | 1.00 (ref) | <.001 |
| ≥40 years | 0.75 (0.68-0.83) |  | 0.77 (0.70-0.85) |  |

Sex and ethnicity included as confounders to generate adjusted hazards ratios.

Includes users with nonmissing age and sex data, and users classified as White or Black/Asian.

**Supplementary Table 4:** Results from cox proportional hazard models reporting associations between sex/age/ethnicity and survival time in programme, excluding users who spent <1 day in the programme

|  | **Unadjusted Model** | | **Adjusted Model** | |
| --- | --- | --- | --- | --- |
|  | **Hazards Ratio**  **(95% CI)** | ***P* value** | **Hazards Ratio (95% CI)** | ***P* value** |
| **Sex** | | | | |
| Male | 1.00 (ref) | <.001 | 1.00 (ref) | <.001 |
| Female | 1.13 (1.07-1.20) |  | 1.11 (1.05-1.18) |  |
| **Age** | | | | |
| <50 years | 1.00 (ref) | <.001 | 1.00 (ref) | <.001 |
| ≥50 years | 0.78 (0.72-0.83) |  | 0.79 (0.73-0.85) |  |
| **Ethnicity** | | | | |
| White | 1.00 (ref) | .10 | 1.00 (ref) | .92 |
| Black/Asian | 1.07 (0.99-1.16) |  | 1.00 (0.92-1.09) |  |

Sex, age, and ethnicity (as appropriate) included as confounders to generate adjusted hazards ratios.

Includes users with nonmissing age and sex data, and users classified as White or Black/Asian.

**Supplementary Table 5:** Retention and engagement metrics by age (categorised as <40 years or ≥ 40 years)

|  | **Median (IQR)** | | | | |
| --- | --- | --- | --- | --- | --- |
|  | **Duration in programme (weeks)** | **Total number of logins** | **Total time spent in programme (mins)** | **Estimated time spent per login (mins)** | **Logins per week** |
| **Age** | | | | | |
| <40 years | 3.00 (0.00-21.00) | 6 (4-13) | 56.57 (18.05-142.27) | 5.15 (2.04-11.59) | 0.90 (0.34-2.33) |
| ≥40 years | 7.57 (0.14-33.79) | 9 (5-19) | 81.43 (27.28-234.19) | 6.40 (2.88-13.55) | 0.84 (0.38-1.89) |
| *P* value | <.001 | <.001 | <.001 | <.001 | .46 |

Excludes users who spent less than one week using the myDESMOND programme

**
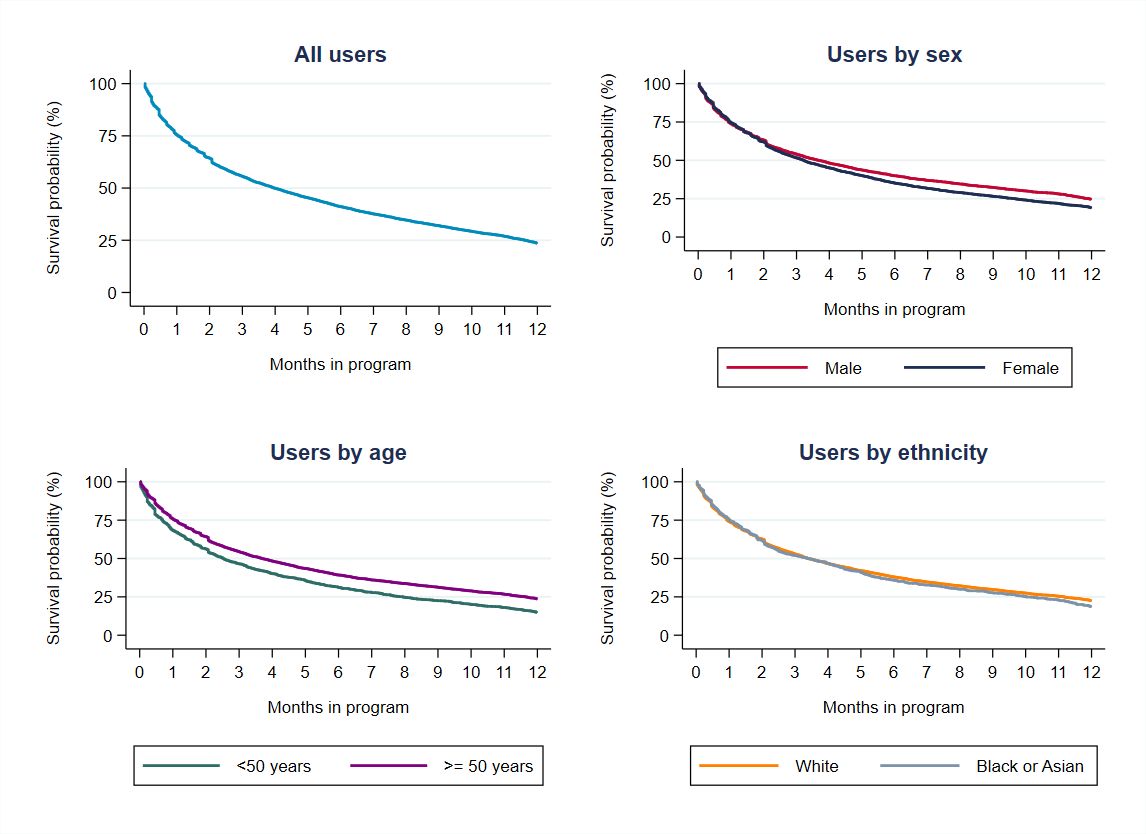
**

**Supplementary Figure 1:** Kaplan-Meier curves showing the time to users stopping using the myDESMOND app after the course of a year, excluding users who spent <1 day in the program, stratified by sex, age, and ethnicity.


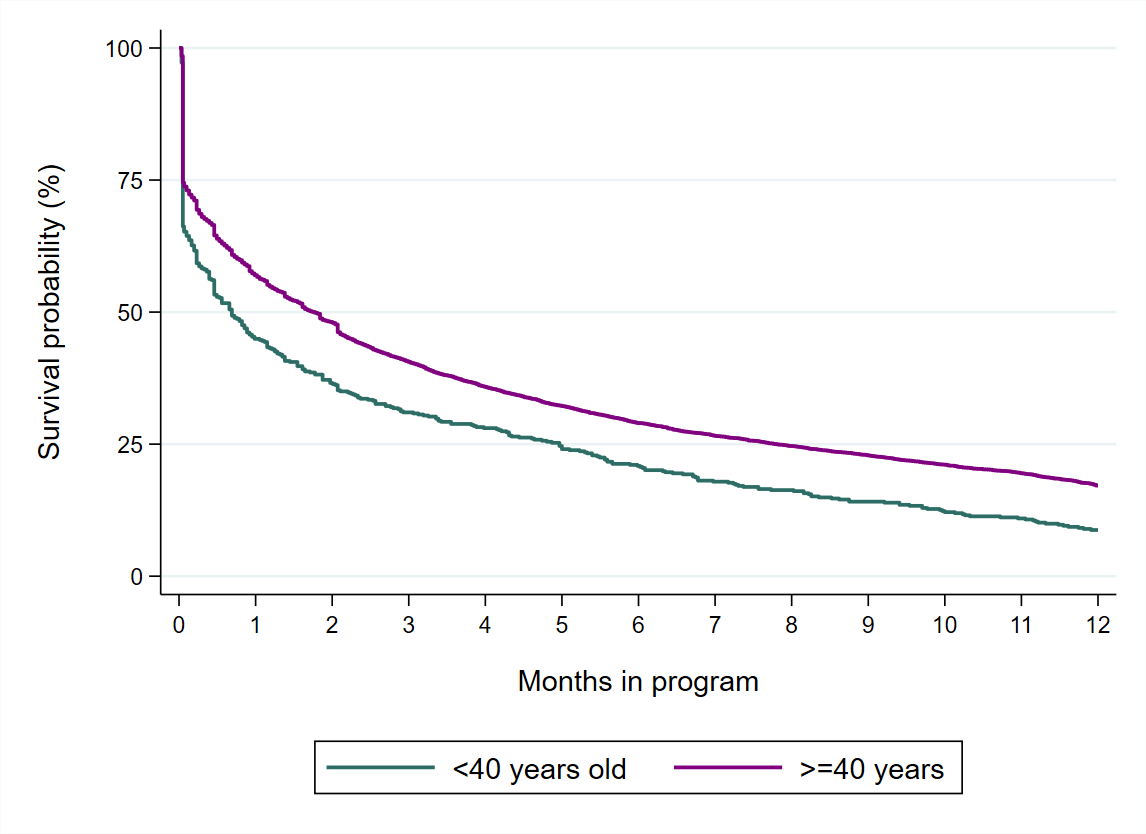


**Supplementary Figure 2:** Kaplan-Meier curves showing the time to users stopping using the myDESMOND app after the course of a year for users stratified by age range (<40 years, ≥40 years)
